# Impact of Rapeseed Press Cake on the Rheological Properties and Expansion Dynamics of Extruded Maize Starch

**DOI:** 10.3390/foods10030616

**Published:** 2021-03-14

**Authors:** Anna Martin, Raffael Osen, Heike Petra Karbstein, M. Azad Emin

**Affiliations:** 1 Department of Food Process Development, Fraunhofer Institute for Process Engineering and Packaging IVV, 85354 Freising, Germany; 2 Singapore Institute of Food and Biotechnology Innovation, Agency for Science, Technology and Research (A*STAR), Singapore 138669, Singapore; Raffael_Osen@sifbi.a-star.edu.sg; 3 Institute of Process Engineering in Life Sciences, Chair of Food Process Engineering, Karlsruhe Institute of Technology, 76131 Karlsruhe, Germany; heike.karbstein@kit.edu (H.P.K.); azad.emin@kit.edu (M.A.E.)

**Keywords:** canola, fiber, expansion, low-moisture extrusion, closed cavity rheometer, biopolymers, plant protein, side stream

## Abstract

Rapeseed press cake (RPC), an oil pressing side product rich in protein and fiber, can be combined with starch and valorized into directly expanded products using extrusion technology. The mechanism of starch expansion has been studied in detail, but the impact of RPC on expansion behavior is poorly understood. However, it can be linked to rheological and physicochemical properties and is a key product quality parameter. Blends with different amounts of RPC (0, 10, 40 g/100 g) were extruded at different barrel temperatures (100, 120, 140 °C) and moisture contents (24 or 29 g/100 g). The initial, intermediate and final sectional, longitudinal and volumetric expansion indices (SEI, LEI, VEI) were monitored directly, 10 s and 24 h after die exit to measure extrudate growth and shrinkage. The viscous and elastic properties of the extruded blends were investigated in a closed cavity rheometer. Starch and blends with 10 g/100 g RPC achieved a high initial SEI followed by significant short-term shrinkage. Blends containing 40 g/100 g RPC did not show any initial expansion. With increasing RPC content, the intermediate SEI decreased, but all samples reached a similar final SEI due to time-dependent swelling of the RPC blends. With increasing RPC content, the elasticity of the starch-based extruded samples significantly increased. Our study shows that comprehensive control and understanding of expansion mechanisms can be achieved only by investigating all stages of extrudate growth and shrinkage. We also found that the closed cavity rheometer is a powerful tool to correlate the rheological properties and expansion mechanisms of biopolymers.

## 1. Introduction

Directly expanded snacks and cereals are often produced using low-moisture extrusion technology [1]. Crucial quality characteristics of such extruded products include the expansion properties that define the texture, giving a certain mouthfeel, bite firmness or crunchiness. Starch is often the basis of extruded blends because its physicochemical and rheological properties support expansion [2]. However, ingredients rich in protein and fiber can be added to enhance the nutritional quality of extruded products [3]. The addition of protein or fiber to starch has been associated with changes in the physicochemical and rheological properties of the melt. The expansion properties are influenced strongly, but can be controlled by adjusting the extrusion process conditions [4,5,6,7].

Press cakes—by-products of the oil or juice pressing process—were previously discussed as candidates for protein and fiber enrichment in foods [8,9]. In a number of studies, press cakes were processed in blends with starch using extrusion technology to generate sustainable and nutritious products. These press cakes significantly influenced the expansion of the products. Whereas sesame, hemp, bilberry, apple and blackcurrant press cakes were shown to reduce the sectional expansion of starch-based extrudates at even low concentrations (<25 g/100 g) [10,11,12,13,14,15], rapeseed press cake (RPC) was shown to increase the sectional and volumetric expansion and reduce the longitudinal expansion when added to potato starch at high concentrations (40–70 g/100 g) [16]. Although the expansion properties could be linked to the rheological properties of the extruded products, the correlations between material, process and product parameters were unclear. This is because press cakes are multicomponent ingredients that make it difficult to attribute changes in product performance to one specific impact factor.

In contrast to the expansion of blends such as starch/press cake, the expansion of pure starch has been described comprehensively and involves several interacting steps [4,17,18,19]. When the hot melt exits the die at the end of the extruder barrel, a pressure drop occurs due to the narrowing of the melt flow channel. The sudden loss of water vapor pressure causes the flash evaporation of any moisture in the melt. Bubble growth occurs, which is driven by the pressure difference between the water vapor bubbles and the surrounding atmosphere [20]. The elasticity, viscosity and surface tension of the melt resists bubble growth [21,22]. When the expansion growth phase is complete, initial shrinkage occurs [22,23], often attributed to the elastic recoil of the matrix. The extrudate is fixed at a certain diameter and porosity due to water evaporation, leading to a continuous decline in the moisture content and temperature, both affecting the glass transition temperature. When the glass transition temperature of the matrix falls, the matrix solidifies. Because expansion is a rapid and dynamic process depending on several variables, it is challenging to describe the expansion mechanism comprehensively.

Typically, expansion is described by expansion indices that are measured and calculated based on the diameter and length of the extrudate in sectional and longitudinal directions without relating it to the time of measurement. The sectional expansion index (SEI) describes the radial growth of the extrudate, the longitudinal expansion index (LEI) describes axial growth and the volumetric expansion index (VEI) is a combination of growth in the radial and axial directions [24]. However, when the properties of extruded products were characterized in previous studies, often only the final SEI was reported and only a few studies, mainly using modeling techniques, investigated extrudate growth and shrinkage separately [23,25].

The experimental studies of Horvat and Schuchmann [26] and Philipp et al. [27] reported sectional expansion separately and investigated initial SEI and final SEI/LEI by imaging the extrudate at the die exit. This revealed that the initial and final expansion of starch-based blends differ, allowing the measurement of extrudate shrinkage. By also measuring the SEI after 24 h, Horvat and Schuchmann [26] observed a second shrinkage phase indicated by a time-dependent decrease in the SEI. Based on the online analysis of the rheological properties of the melt, the elastic and viscous properties of the melt were presumed to be associated with the initial growth of the extrudate, whereas the glass transition temperature was correlated with the final fixation of the extrudate. Shrinkage is only discussed in a limited number of previous studies and is often underestimated in prediction models, especially in extrusion processes where the moisture content is > 25 g/100 g [26].

To control all the expansion steps when protein/fiber-rich ingredients are added to starch, a comprehensive understanding of material and process parameters during extrusion is required. This is necessary to develop new products with designated product characteristics. The addition of RPC to starch is likely to influence the growth and shrinkage of the extrudate, a time-dependent mechanism that can be controlled by the moisture content and barrel temperature. Accordingly, we investigated the effect of two concentrations of RPC on the effect of starch expansion, including growth and shrinkage, compared to pure starch. We tested three relevant barrel temperatures and two moisture contents in order to monitor the impact of process conditions on expansion. Expansion indices were monitored directly after die exit by camera imaging, then 10 s later and 24 h later. Moreover, the rheological properties of the extruded samples were investigated under extrusion-like conditions in a closed cavity rheometer. The impact of thermomechanical treatment during extrusion on the elastic and viscous properties of the blends was observed and correlated to growth and shrinkage during expansion. Furthermore, we investigated the extruder response, indicated by the specific mechanical energy input (SME) as a measure of shear stress and the product temperature as a measure of thermal energy input.

## 2. Materials and Methods

### 2.1. Raw Materials

Cold-pressed fiber-reduced rapeseed press cake (RPC) was kindly provided by Teutoburger Ölmühle (Ibbenbüren, Germany). After grinding the RPC using a Hosokawa 100 UPZ mill (Hosokawa Alpine, Augsburg, Germany) at 800/min equipped with a 0.5-mm mesh screen, the particle size distribution (D_v0.5_) was 261.1 ± 4.5 µm. Rapeseed peel was separated (sieving and sifting) from RPC by the manufacturer, reducing the fiber content compared to standard RPC varieties. Maize starch (MS) was provided by Ingredion (Hamburg, Germany) and the D_v0.5_ was 17.7 ± 0.14 µm. MS and RPC were investigated as single ingredients and RPC/starch blends with ratios of 40/50 and 10/70 g/100 g wet basis (w.b.). To achieve equal lipid (15.6 ± 0.6) and raw fiber (3.3 ± 0.1) contents (g/100 g dry matter (d.m.) basis) in all blends, 5–10 g/100 g (w.b.) rapeseed peel and oil (Teutoburger Ölmühle, Ibbenbüren, Germany) was added, giving blend compositions of 40/50/5/5 and 10/70/10/10 RPC/starch/peel/oil [16]. The rapeseed peel D_v0.5_ was 418.9 ± 15.9 µm.

### 2.2. Chemical Analysis and Functional Properties

The moisture content of the materials and extrudates was determined according to the German Food Act [28]. The protein content was analyzed based on the Dumas method according to the German Food Act [28] using a TruMac N Protein Analyzer (LECO, St. Joseph, MI, USA). The ash content was determined according to AOAC International method 945.46 [29]. The crude fiber content was determined according to AOAC International method 962.09 [30]. The starch content was determined as previously described [31]. Water absorption (g/g) of the raw materials and extruded samples was analyzed according to AACC method 56–20.01 and water solubility (%) was determined as previously described [32]. The particle size of MS, RPC and RP was determined using a Malvern Mastersizer S Long Bed Version 2.15 laser diffraction particle size analyzer (Malvern Instruments, Malvern, UK) as previously described [33].

### 2.3. Low-Moisture Extrusion

A co-rotating twin-screw ZSK26Mc extruder (Coperion, Stuttgart, Germany) with a L/D ratio of 25/1 was used for the preparation of expanded products. The extrusion process was carried out at 10 kg/h constant mass flow rate of dry feed (m_dry_) and a screw speed of 300 rpm [16]. The temperature of the last barrel segment (T_Barrel_) was set to 100, 120 or 140 °C and the moisture content of the melt (M_melt_) was kept constant at 24 or 29 g/100 g d.m. A round orifice die was used with a diameter of 4.5 mm. After extrusion, samples were dried in a Thermo Scientific Heraeus UT 6760 hot air oven (Thermo Electron LED, Langenselbold, Germany) at 40 °C for 24 h and ground to <0.5 mm particle size using a Grindomix GM 200 knife mill (Retsch, Haan, Germany). Extruder responses (pressure at die exit, torque and product temperature) were monitored throughout the sample-taking period. The specific mechanical energy (SME) was calculated as previously described [4,34]. Extrusion trials were carried out in duplicate.

### 2.4. Expansion Properties

**Figure 1:**
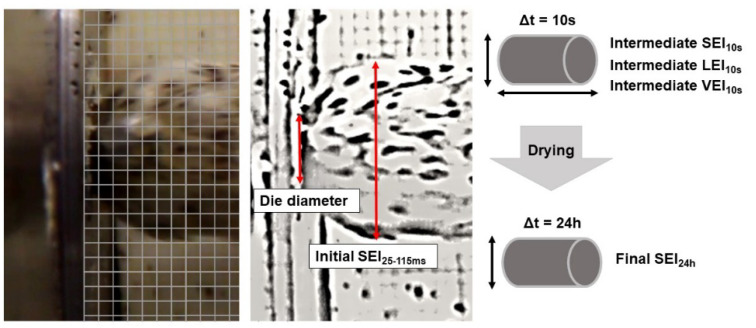
Representative image of extrudate expansion and the method used to determine expansion parameters.

Growth and shrinkage of extruded products in the radial and axial directions are often expressed as sectional (SEI), longitudinal (LEI) and volumetric (VEI) expansion indices. However, to account for all three main phases of expansion (growth directly after die exit, first shrinkage and second shrinkage), new expansion indices have been defined. The initial sectional expansion was evaluated according to Horvat and Schuchmann [26] with modifications. They proposed that the initial growth phase of starch-based extrudates was finalized after 25–115 ms. Therefore, the initial sectional expansion was given as SEI_25–115ms_. A CHDHX-801-RW 4K60 camera (GoPro, San Mateo, CA, USA) was placed at the extruder die exit and the initial diameter of extrudate strains was determined via video image analysis based on at least 20 images per recording. During recording, a scale paper was placed at the die exit to determine the sectional diameter growth (Figure 1). The initial sectional expansion (SEI_25–115ms_) was then calculated based on the extrudate diameter (D_E_) and die diameter (D_D_) using Equation (1).

(1) $$\mathrm{SEI}={\left(\frac{D_{E}}{D_{D}}\right)}^{2}$$

Intermediate sectional expansion (SEI_10s_) was determined by measuring the diameter of extrudate strains with a digital caliper exactly 10 s after exiting the die. At least 10 extrudate strains were measured. Furthermore, the weight (gravimetrically) and length (digital caliper) of extrudate strains taken over a period of 10 s was monitored in order to calculate the intermediate longitudinal (LEI_10s_) and volumetric expansion (VEI_10s_). Longitudinal expansion is usually described as the ratio of the velocity of extrudate (v_E_) after die exit and the velocity of the melt (v_M_) inside the die (Equation (2)).

(2) $$\mathrm{LEI}=\frac{v_{E}}{v_{M}}$$

v_M_ can be calculated from the total given feed rate (${\dot{Q}}_{M}$) and the cross-sectional area of the die (S_die_) using Equation (3). The density of the melt was presumed to be 1.400 kg/m^3^ as reported in previous studies [35].

(3) $$v_{M}=\frac{{\dot{Q}}_{M}}{S_{\mathrm{die}}}$$

The intermediate volumetric expansion (VEI_10s_) was calculated as the product of SEI_10s_ and LEI_10s_. Final sectional expansion (SEI_24h_) of the extrudate strains was determined using a digital caliper after drying the extrudates for 24 h at 40 °C. At least 10 extrudate strains representing each blend and process setting were analyzed. The dry matter of the dried extrudates was measured according to the German Food Act [28]. The samples were milled to <500 µm for further analysis.

### 2.5. Rheological Properties

For thermomechanical treatment, we used an RPA elite closed-cavity rheometer (TA Instruments, New Castle, DE, USA). Because the cavity can be pressurized (4.5 MPa) and sealed, the device allows the analysis of low-moisture samples at elevated temperatures without water vaporization or slippage [36]. Before each test, the moisture content of dried and milled extruded samples was determined [28] and adjusted to 24 or 29 g/100 g (d.m.) by mixing the powdered materials with deionized water in a Thermomix (Vorwerk, Wuppertal, Germany). To ensure homogenous water distribution, samples were incubated in a refrigerator (4 °C) for at least 8 h. For rheological analysis, samples were brought to room temperature and 6.0 ± 0.1 g samples were placed on the cone for each test. Each test was carried out at least in duplicate. The impact of extrusion on the rheological properties of the samples was evaluated by treating extruded samples for 10 s at a shear rate of $\dot{\gamma }$_Pre_ = 31 s^−1^, before maintaining a constant measurement shear rate of $\dot{\gamma }$_M_ = 0.1 s^−1^ for 8 min (corresponding to a deformation of 1% and a frequency of 1 Hz). The pre-treatment and measurement temperatures (T_Pre_ and T_M_) were equally set to 100, 120 or 140 °C, corresponding to the barrel temperature (T_Barrel_) applied during extrusion. The rheological parameters storage and loss modulus G’ and G’’ were calculated based on the torque recorded by a transducer in the upper cone of the device [37].

## 3. Results

### 3.1. Chemical Composition

Table 1 shows the chemical composition of RPC, RP, MS and the blends. The protein content of RPC was significantly higher compared to that of RP, but the raw fiber content and particle size were significantly lower. The starch content of RPC and RP was low and the purity of MS was very high, as indicated by the starch content of >99 g/100 g. With increasing RPC content, the protein and starch content of the blends decreased.

### 3.2. Expansion

#### 3.2.1. Sectional Expansion

**Figure 2:**
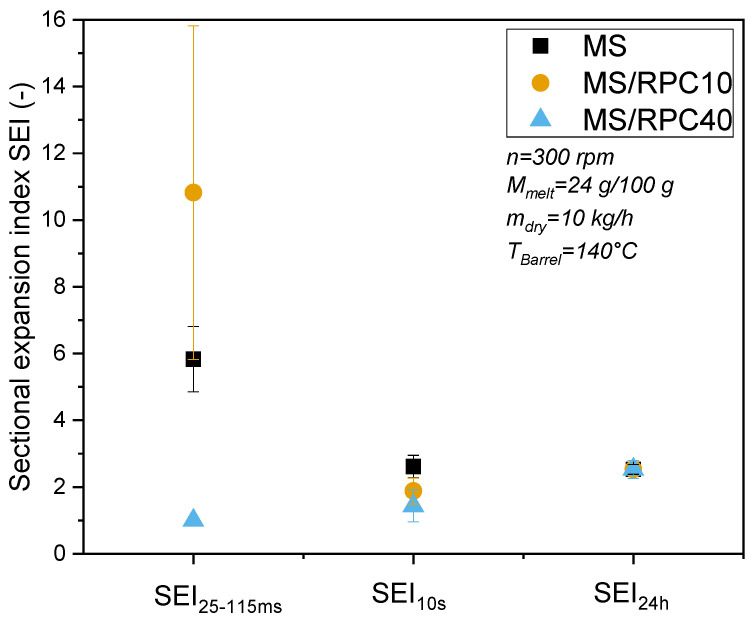
Initial, intermediate and final sectional expansion indices (SEI_25–115ms_, SEI_10s_, SEI_24h_) of maize starch (MS) extruded as a pure component or in blends with 10 or 40 g/100 g (w.b.) rapeseed press cake (RPC). Samples were processed at a barrel temperature (T_Barrel_) of 140 °C, a mass flow rate (m_dry_) of 10 kg/h, a moisture content (M_melt_) of 24 g/100 g (d.m.) and a screw speed (n) of 300 rpm.

Figure 2 shows the effect of RPC on the initial, intermediate and final SEI of extruded MS and MS/RPC blends. The SEI_15–25ms_ of MS was significantly higher than the SEI_10sec_ and SEI_24h_ at T_Barrel_ = 140 °C, but there was no significant difference between SEI_10sec_ and SEI_24h_. When 10 g/100 g RPC was added to the starch, the SEI_15–25ms_ was significantly higher than the SEI_10s_ and SEI_24h_. However, when 40 g/100 g RPC was present, no initial expansion was observed, but the SEI increased over time and was highest after 24 h.

**Figure 3:**
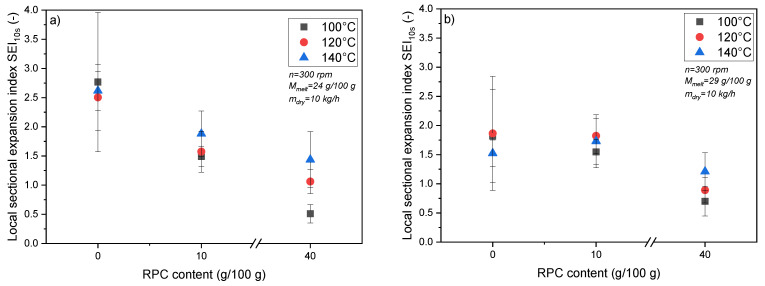
Intermediate sectional expansion index (SEI_10s_) of maize starch (MS) extruded as a pure component or in blends with 10 or 40 g/100 g (w.b.) rapeseed press cake (RPC). Samples were processed at a barrel temperature (T_Barrel_) of 100, 120 or 140 °C, a mass flow rate (m_dry_) of 10 kg/h, a moisture content (M_melt_) of (**a**) 24 g/100 g (d.m.) or (**b**) 29 g/100 g (d.m.) and a screw speed (n) of 300 rpm.

Figure 3 shows the impact of RPC on the SEI_10s_ of MS and MS/RPC blends extruded with a moisture content of 24 g/100 g (Figure 3a) or 29 g/100 g (Figure 3b). MS achieved the highest SEI_10s_ regardless of T_Barrel_ whereas the addition of RPC caused the SEI_10s_ to decrease. However, the SEI_10s_ of MS declined as the moisture content increased, whereas the moisture content had a negligible effect on the SEI_10s_ of blends containing RPC. In the MS/RPC40 blend, the SEI_10s_ increased along with the T_Barrel_ and the effect was more pronounced at the lower moisture content. A similar phenomenon was observed for MS/RPC10 when the moisture content was 24 g/100 g. The variability of SEI_10s_ for MS extruded at T_Barrel_ = 100 °C can be attributed to the irregular oscillating surface of the extrudates. This effect has been described as the “shark skin” phenomenon and can be attributed to stick-slip mechanisms between the melt and the inner surface of the die.

#### 3.2.2. Longitudinal and Volumetric Expansion

**Figure 4:**
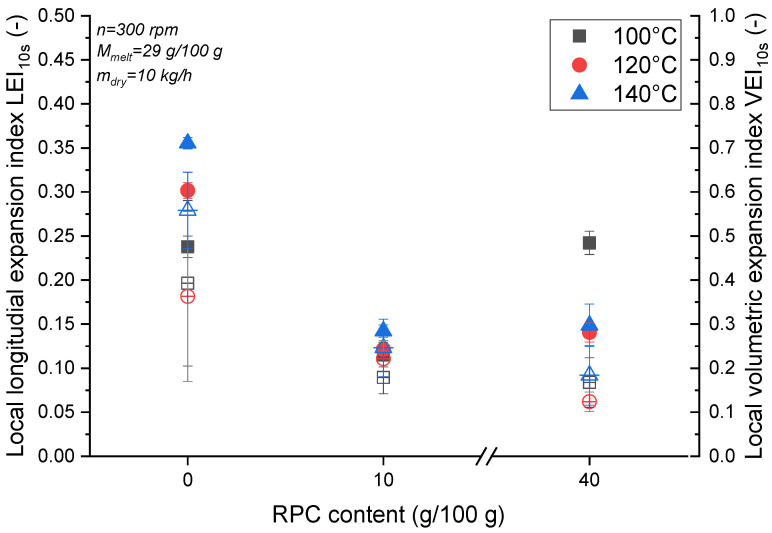
Effect of the barrel temperature (T_Barrel_ = 100, 120 or 140 °C) and rapeseed press cake (RPC) content (0, 10 or 40 g/100 g (w.b.)) on maize starch (MS) extruded at 29 g/100 g (d.m.) moisture content. RPC is plotted against intermediate longitudinal expansion (LEI_10s_, filled symbols) and intermediate volumetric expansion (VEI_10s_, empty symbols).

Figure 4 shows how RPC influences the LEI_10s_ and VEI_10s_ of MS and MS/RPC blends at different T_Barrel_ values. When the moisture content during extrusion was 29 g/100 g at a T_Barrel_ of 120 or 140 °C, the LEI_10s_ and VEI_10s_ of MS were higher than the corresponding values for the RPC blends. At T_Barrel_ = 100 °C, MS and MS/RPC40 were similar in terms of LEI_10s_, but the LEI_10s_ of MS/RPC10 was lower. Furthermore, MS/RPC10 and MS/RPC40 were similar in terms of VEI_10s_ but MS achieved a higher VEI_10s_ than MS/RPC10 and MS/RPC40. With increasing T_Barrel_, the LEI_10s_ of MS increased and the VEI_10s_ of MS peaked at T_Barrel_ = 140 °C. For MS/RPC10, T_Barrel_ had a negligible effect on the LEI_10s_ and VEI_10s_. For MS/RPC40, the highest LEI_10s_ was observed at T_Barrel_ = 100 °C and decreased with increasing T_Barrel_, but did not differ when comparing T_Barrel_ values of 120 and 140 °C. The VEI_10s_ of MS/RPC40 was not significantly influenced by T_Barrel_.

### 3.3. Impact of Extrusion Treatment on Viscous and Elastic Properties of Starch/RPC Blends

**Figure 5:**
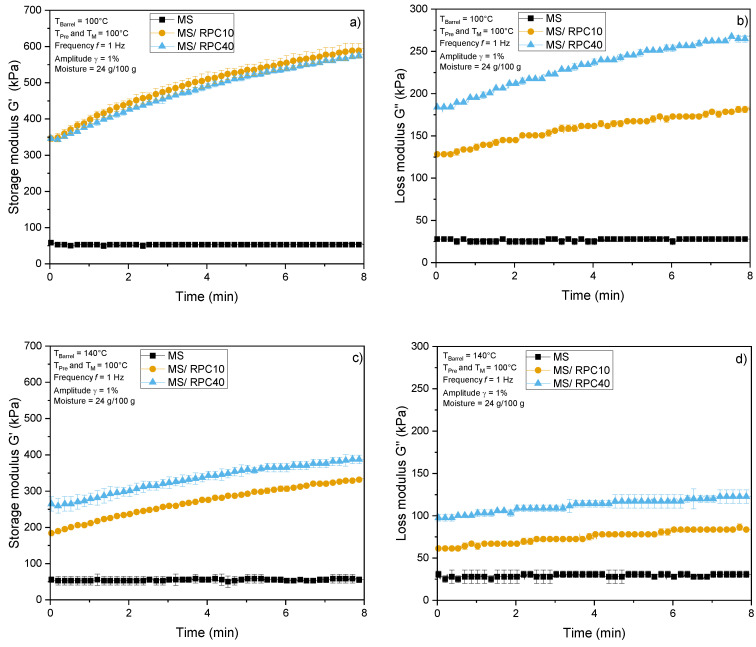
Storage modulus G’ (**a**,**c**) and loss modulus G’’ (**b**,**d**) (kPa) as a function of treatment time (min), pre-treatment and measurement temperature (T_Pre_, T_M_) of maize starch (MS) and maize starch blended with 10 or 40 g/100 g (w.b.) rapeseed press cake (RPC) rehydrated to a moisture content of 24 g/100 g (d.m.). The samples were extruded at a moisture content of 24 g/100 g (d.m.) and barrel temperatures (T_Barrel_) of 100 or 140 °C.

Figure 5 shows the impact of time, pre-treatment and measurement temperature (T_Pre_, T_M_) on the viscous and elastic properties of MS and MS/RPC blends extruded at various barrel temperatures. Due to flash evaporation, we assume that the product temperature (Section 3.4) rapidly declines to 100 °C after die exit. Therefore, a measurement temperature of 100 °C was applied to all samples to investigate the rheological properties of the melts immediately after leaving the die.

At a T_Pre_ and T_M_ of 100 °C, the G’ and G’’ of MS extruded at 100 or 140 °C did not change over time, whereas the G’ and G’’ of MS/RPC10 and MS/RPC40 were far higher compared to MS and the curve progression indicated that G’ increases over time. In MS/RPC10 and MS/RPC40, the increase in G’ and G’’ was higher when the T_Barrel_ was 100 °C rather than 140 °C. Increasing the T_Pre_ and T_M_ to 140 °C reduced the G’ and G’’ of all samples and the G’ and G’’ of MS were again lower than the corresponding values for MS/RPC10 and MS/RPC40. The G’ curve progression was similar for MS/RPC10 and MS/RPC40, except when T_Barrel_ was set to 140 °C and T_Pre_ and T_M_ were set to 100 °C, in which case MS/RPC40 achieved a higher G’ than MS/RPC10. MS/RPC40 achieved a significantly higher G’’ than MS/RPC10 at all temperatures.

**Figure 6:**
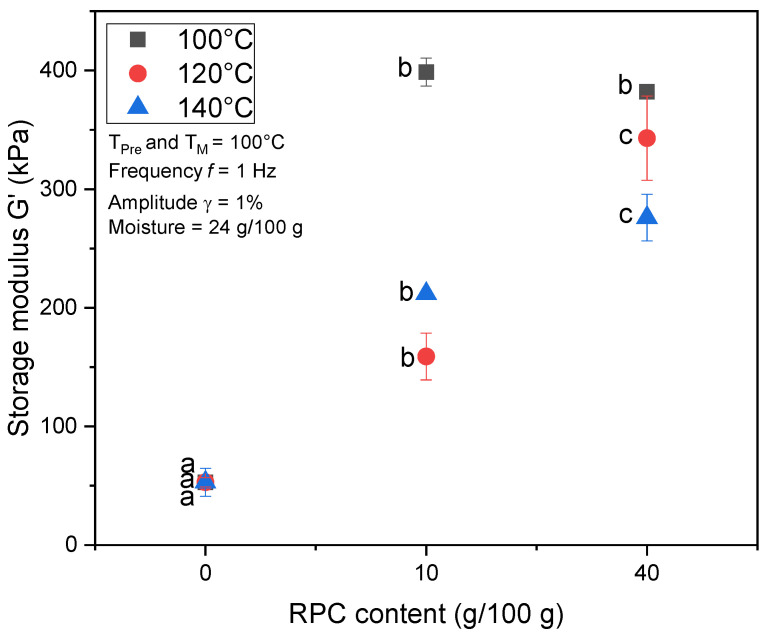
Storage modulus G‘ (kPa) after 1 min treatment time (T_T_) as a function of rapeseed press cake (RPC) content (0, 10 or 40 g/100 g (w.b.)) and barrel temperature (T_Barrel_ = 100, 120, 140 °C) of extruded maize starch (MS) blends. Samples extruded and rehydrated to 24 g/100 g (d.m.) moisture content; pre-treatment and measurement temperature (T_Pre_, T_M_) 100 °C. Mean values with different superscript letters indicate significant differences (*p* < 0.05) based on a one-way analysis of variance (ANOVA). Means were compared using Tukey’s honest significance test.

Figure 6 shows the G’ of MS and MS/RPC blends as an effect of T_Barrel_ after measurement for 1 min at 100 °C. T_Barrel_ did not affect the G’ of MS, but G’ decreased with increasing T_Barrel_ in the RPC blends. Increasing the T_Barrel_ from 100 to 120 °C caused a decline in G’ for both MS/RPC10 and MS/RPC40, but this effect was significantly greater for MS/RPC10. The addition of RPC generally caused G’ to increase significantly regardless of T_Barrel_. At T_Barrel_ = 100 °C, there was no significant difference between the G’ of MS/RPC10 and MS/RPC40.

### 3.4. Extruder Response

Table 2 summarizes the extruder responses (pressure at die, product temperature, torque and SME) as a function of RPC content and T_Barrel_. At T_Barrel_ = 100 and 120 °C, the pressure at the die and product temperature increased in the order MS, MS/RPC40, MS/RPC10. At T_Barrel_ = 140 °C, MS reached the highest product temperature and MS/RPC10 reached the highest pressure at the die (followed by MS) whereas MS/RPC40 reached a comparatively low pressure at the die. The SME, a function of torque, was highest for MS and decreased significantly (*p* < 0.05) with increasing RPC content regardless of T_Barrel_.

The high SME of MS compared to RPC blends can be attributed to the less compact structure generated by fibrous RPC components, which have a larger particle size than MS, distributed in the starch matrix. These insoluble fibers may also explain the higher pressure at the die of MS/RPC10 compared to MS and MS/RPC40. The fibers might be solubilized during extrusion, resulting in a high water binding and holding capacity leading to swelling and a higher pressure build up at the die exit (Section 4.1 and Section 4.4). The dry matter content of extruded samples is shown in Table 3 as a function of the applied moisture content during extrusion. Due to the drying of the extrudates after extrusion, the dry matter content of all samples was >90 g/100 g. Regardless of T_Barrel_, samples extruded at lower moisture contents featured higher dry matter contents after drying.

### 3.5. Water Absorption Index and Water Solubility Index

**Figure 7:**
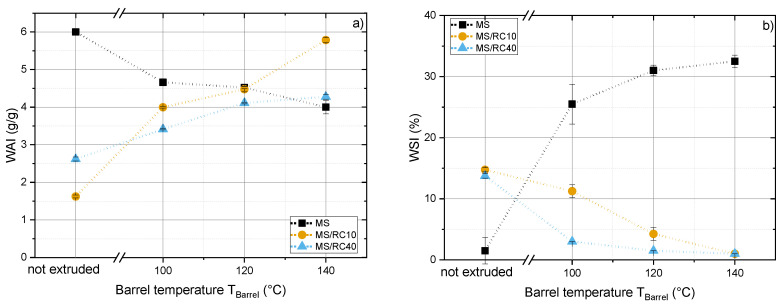
Water absorption and solubility. (**a**) Water absorption index (WAI) and (**b**) water solubility index (WSI) as a function of barrel temperature (T_Barrel_ = 100, 120, 140 °C) of maize starch (MS) blended with 10 or 40 g/100 g (w.b.) rapeseed press cake (RPC) extruded at 29 g/100 g (d.m.) moisture content. Lines have been added for visual clarity.

The water absorption and water solubility indices (WAI and WSI) of non-extruded and extruded starch and starch/RPC blends are compared in Figure 7. The WAI and WSI of starch-based blends were highly dependent on the extrusion process conditions. Overall, the WAI of all samples was relatively low. The WAI of MS was highest for the non-extruded samples and decreased with increasing T_Barrel_, whereas the WSI was lowest for the non-extruded samples and increased with increasing T_Barrel_. The WAI and WSI of RPC blends showed the opposite trend. The WAI of RPC blends was low for the non-extruded samples and increased with increasing T_Barrel_, whereas the WSI was highest for the non-extruded samples and declined with increasing T_Barrel_. These effects probably reflect the presence of rapeseed fiber and protein in the RPC blends as discussed in Section 4.4.

## 4. Discussion

### 4.1. Correlation between Sectional Expansion Properties and Rheological Properties

The rheological properties of blends play an important role in extrudate growth and shrinkage [6,17,38]. Whereas the storage modulus represents the elastic properties of a material, the loss modulus is linked to the viscous properties. The structuring of materials during expansion has been correlated to elastic material properties in previous studies [6,17,38,39]. The addition of RPC changed the viscous and elastic properties of the starch- based blends (Section 3.3), specifically affecting the pressure and the product temperature at the die exit during extrusion (Table 2). These parameters synergistically affect not only the degree of expansion, but the direction (radial, axial) and preservation (shrinkage) of expansion (Figure 2).

RPC blends extruded at T_Barrel_ = 100–140 °C achieved greater elasticity than MS, possibly reflecting the intermediate expansion, SEI_10s_ (Figure 5 and Figure 6). The G’ of MS/RPC10 was higher than that of MS, resulting in greater elastic recoil and more pronounced shrinkage, thus, in turn, reducing the SEI_10s_. Furthermore, the G’ of MS/RPC40 extruded at T_Barrel_ = 140 °C was significantly higher than that of MS/RPC10 after 1 min treatment time at 100 °C (Figure 6). This might explain the large difference in SEI_25–115ms_ between blends containing 10 and 40 g/100 RPC. A certain amount of elasticity is required to allow bubble growth and stabilize the vapor bubbles in the matrix, as seen for MS/RPC10. However, previous studies revealed that high extensional forces are caused by the pressure drop at the extruder barrel; thus, the vapor bubbles are subjected to forces beyond their elastic limits [40]. Accordingly, blends containing 40 g/100 g RPC exceeded the threshold for vapor bubble stabilization in the melt. Blends containing 10 or 40 g/100 g RPC contained equal amounts of lipid and raw fiber, but the protein content increased in line with the RPC content. The increase of G’ and G’’ over time was highest when the blends were extruded at T_Barrel_ = 100 °C (Section 3.3). This indicates that at T_Barrel_ = 100 °C the rapeseed proteins in the blends are not fully polymerized and are still reactive after extrusion treatment. This may explain the more pronounced effect of T_Barrel_ on the SEI_10s_ of MS/RPC40.

In addition to the effect of rapeseed proteins on the initial expansion process, the lower SEI_10s_ of RPC blends compared to MS may reflect the presence of insoluble dietary fiber originating from the RPC. Previous results show that once a critical concentration of insoluble fibers has been reached in extruded blends, sectional expansion is often limited, although this has only been investigated based on the final SEI [41]. The fibers originating from RPC can align themselves in the direction of flow, strengthening the expanding melt, increasing its mechanical resistance in the axial direction and creating an anisotropic matrix structure [42]. This in turn can limit sectional expansion, because the structural anisotropy inhibits the biaxial extensional properties of the extruded blends [21]. A lower SEI_10s_ in the presence of RPC (Figure 3) agrees with previous studies investigating the effect of oilseed and juice press cakes on sectional expansion [10,11,12,13,14,43,44]. However, these studies monitored the final SEI of the extrudates at an unknown time point, so the results cannot be compared directly and, in our study, may be linked to SEI _24h_.

The high SEI_25–115ms_ of MS/RPC10 (Figure 2) may also be linked to the presence of insoluble fiber. The raw fiber content of MS/RPC10 and MS/RPC40 was kept constant by adding 10 and 5 g/100 g (w.b.) rapeseed peel, respectively (Section 2.1). Rapeseed peel consists of >80 g/100 g (d.m.) insoluble dietary fiber [16], so MS/RPC10 contains higher amounts of insoluble fiber than MS/RPC40. Previous studies reported that the addition of insoluble fiber (i.e., wheat bran, corn bran, brewer’s spent grain, cauliflower, soy and sugar beet fiber) led to cellular structures with smaller air cell sizes but a higher cell density [41]. The authors attributed this effect to more pronounced nucleation in the extruder. Therefore, we assume that insoluble rapeseed fibers in MS/RPC10 promoted nucleation and a consequently rapid expansion directly after die exit. Vapor-driven expansion is predominantly influenced by expansion in the sectional direction and has been used as an indirect measure of bubble growth rate [23]. Moreover, a large amount of insoluble dietary fiber, as found in MS/RPC10, may promote the swelling of the extrudate at the die exit due to the solubilization of insoluble fibers [45]. This would increase the moisture binding capacity of the melt and the SEI_25–115ms_. The high water absorption of MS/RPC10 extruded at T_Barrel_ = 140 °C supports these assumptions (Section 3.5 and Section 4.4).

The effect of fibers on sectional expansion appears to be concentration dependent and strongly influenced by the applied process conditions [21]. In previous studies, the addition of fiber to materials with a low moisture content resulted in the significant restriction of final sectional expansion. However, when the moisture content was higher (>20 g/100 g), as in the current study, the presence of fibers in the blends had a limited effect on the final expansion ratio [46]. The similar SEI_24h_ of MS and RPC blends supports these findings. The large SEI_25–115ms_ of MS/RPC10 extruded at T_Barrel_ = 140 °C was accompanied by a lower mechanical energy input, a lower product temperature and a higher pressure at the die compared to MS and MS/RPC40 (Section 3.4). An increase in pressure due to a lower SME and product temperature has been reported previously and may have encouraged the more pronounced initial radial expansion of MS/RPC10 [47].

Although the stages of expansion are described elsewhere as exclusively growth followed by shrinkage [26], the RPC blends in our study increased in diameter within the time of 10 s and 24 h after die exit. This effect was probably initiated by the high swelling potential of rapeseed fibers, allowing them to bind the moisture in the melt during processing and leading to a gradual increase in the diameter of the extrudate strains over time. Thus far, the effect of extrudate shrinkage during post-processing (e.g., drying and storage) has received little attention from researchers and is underestimated in expansion prediction models, although it is a critical parameter for final product quality [26].

### 4.2. Sectional Expansion Properties and Glass Transition Temperature

The observed expansion properties of extruded starch and starch/RPC blends may also be related to the glass transition temperature (T_G_) of the blends. Intermediate sectional expansion, determined by measuring the extrudate strain diameter 10 s after die exit, can be linked to exceeding the T_G_ required for the solidification of the matrix [27,48]. The higher SEI_10s_ of MS compared to RPC blends can be linked to a possible decrease in T_G_ due to the addition of RPC.

The T_G_ of maize starch is 20–60 °C with a moisture content of 25–50 g/100 g [49]. Gelatinized starch, generated by thermomechanical treatment during extrusion, has an even lower T_G_ of <15 °C with a moisture content >25 g/100 g [49]. The T_G_ of several plant proteins has also been reported. Barley proteins have a T_G_ of 30 °C with a moisture content of 15 g/100 g [50]. Gluten was reported to show a T_G_ of 70 °C at a moisture content of 12.5 g/100 g, as determined by differential scanning calorimetry, whereas dynamic mechanical thermal analysis of gluten conditioned to >25 g/100 g moisture content resulted in a T_G_ of <10 °C [51]. The T_G_ of soy protein was ~40 °C at a moisture content of 15 g/100 g [51] and freeze-dried canola protein isolates had a T_G_ of 50–65 °C [52].

When plant-based components were added to starch in extrusion studies, the T_G_ decreased in line with the content of plant material, as seen for pea starch blends with an increasing pea protein content [53]. Based on these findings, it is likely that the addition of RPC led to a lower T_G_ compared to starch (possibly below room temperature), such that no complete solidification of RPC extrudates occurred before drying. MS, presumably, passed the T_G_ and solidified when it was brought to room temperature shortly after die exit, resulting in no significant difference between the SEI_10s_ and SEI_24h_. Although room temperature was ~23 °C during extrusion, previous studies suggest that the first stage of solidification of extrudates begins at 30–45 °C above T_G_ [26].

Generally, T_G_ decreases with increasing moisture content. During drying, constant moisture loss increased the T_G_ of RPC blends such that the T_G_ may have been reached at an unknown point during the 24 h period, explaining the increased diameter of MS/RPC10 and MS/RPC40 extrudates over time. The lower T_G_ with increasing moisture contents also explains the lower SEI_10s_ of MS with increasing moisture content. With a higher T_G_, the extrudate solidifies at an earlier stage of shrinkage and a larger diameter.

In previous studies, the first shrinkage of starch was attributed to elastic recovery or water vapor condensation inside a vapor cell, generating a negative pressure difference. In parallel, the sudden moisture loss leads to a decrease in temperature, which in turn increases the viscosity of the matrix. During drying, further moisture loss and lower temperatures were reported to facilitate the crossing of the T_G_ to initiate a second decrease in the extrudate diameter [26]. However, we did not observe a second shrinkage stage for MS in this study, which can be attributed to differences in the chemical composition and physicochemical material properties compared to previous studies. The starch content of the MS we used was 99.06 ± 0.01 g/100 g, whereas Horvat and Schuchmann [26] used corn grits with a starch content of 58 g/100 g. Therefore, the T_G_ as well as elastic and viscous properties may differ considerably resulting in a different shrinkage mechanism.

### 4.3. Correlation between Longitudinal Expansion and Rheological Properties

The longitudinal expansion properties of starch-based melts have been associated with their viscous properties [17,24,54]. The viscosity of the melt in turn affects the pressure at the die. The lower LEI_10s_ of MS/RPC10 extruded at 29 g/100 g was accompanied by a higher SEI_25–115ms_, in agreement with previous reports [55]. At T_Barrel_ = 100 °C, MS/RPC10 generated a higher pressure at the die than MS/RPC40 and MS, which behaved similarly to each other (Section 3.4). A low pressure at the die is related to increased longitudinal expansion, because a shallow pressure gradient between the inside and outside of the barrel achieves the water vapor pressure shortly after die exit and no distinct bubble growth is observed [56].

The higher LEI_10s_ of MS/RPC40 compared to MS/RPC10, especially when samples were extruded at T_Barrel_ = 100 °C, is linked to the rheological properties of the blends. We were also able to relate longitudinal expansion to the viscous properties of the melt, as previously reported [17]. Furthermore, the G’’ of extruded MS/RPC40 was far larger than that of MS and MS/RPC10, presumably leading to the higher LEI_10s_ (Section 3.3). This effect was more pronounced when the barrel and measurement temperature was 100 °C rather than 140 °C.

The higher LEI_10s_ of MS with increasing T_Barrel_ can be attributed to the effect of T_Barrel_ on viscosity. A higher T_Barrel_ reduces the viscosity of the melt, allowing the melt to reach a higher velocity outside the die and thus encouraging longitudinal expansion. High radial expansion, as seen for MS with a lower moisture content, reduces the velocity of the melt outside the die, leading to a comparatively small LEI.

### 4.4. Water Absorption and Water Solubility

Some studies report that the WAI of starch increases with thermomechanical treatment due to gelatinization, the breakage of intramolecular and intermolecular bonds and the exposed hydroxyl groups that can form hydrogen bonds with water [57]. However, this effect was not observed in our study. We assume that the relatively high moisture content in our study generated resistant starch due to the better nucleation and elongation of amylose and amylopectin chains. This effect can induce recrystallization or retrogradation leading to the formation of hydrogen bond-stabilized dense starch structures, reducing the WAI of extruded starch [58].

The increasing WAI of RPC blends can be attributed to the solubilization of insoluble dietary fiber by thermomechanical treatment. A high water binding capacity for extruded fiber-rich biopolymers was also reported in previous studies of lupin fiber [45], wheat bran [59], orange pomace [60], carrot residues [61] and barley meal [62]. Fetzer et al. [63] reported cellulose, hemicellulose and lignin contents of 6.8 ± 0.6, 3.9 ± 0.6 and 11.4 ± 0.1 g/100 g d.m., respectively, for cold-pressed RPC. The solubilization of hemicellulose and pectin-like polymers, which are present in RPC, was particularly well correlated with higher water-binding capacity [64,65].

The lower WSI of RPC blends compared to starch may reflect the presence of rapeseed proteins. At T_Barrel_ = 100 and 120 °C, the WSI of MS/RPC10 was higher than that of MS/RPC40. This indicates that the larger protein content of MS/RPC40 may encourage rapeseed proteins that are soluble in their native state to denature, unfold and form new bonds due to thermomechanical treatment, which reduces their solubility in water.

The increasing WSI of MS with increasing T_Barrel_ may be related to the separation of amylose and amylopectin chains from an extendable matrix, thus increasing the solubility [57]. Previous studies reported a higher WSI for fully gelatinized MS and indicated that a moisture content of 28–30 g/100 g during extrusion promotes a maximum degree of gelatinization [66]. Therefore, we assume that the process conditions in our study led to the full gelatinization of starch resulting in a higher WSI.

## 5. Conclusions

In this study, we used image processing to investigate the expansion dynamics (initial, intermediate and final expansion) of starch-based blends enriched with RPC, in order to determine the effect of different RPC contents and process conditions on the rheological properties, expansion, extruder response and physicochemical parameters of directly expanded products. We tested two RPC contents (10 and 40 g/100 g) in addition to pure MS at three barrel temperatures (100, 120, 140 °C) and two moisture contents (24 and 29 g/100 g). A closed cavity rheometer simulating extrusion conditions was used to investigate the rheological properties of the starch/press cake blends after extrusion.

The expansion of starch was initially high but was dominated by severe shrinkage shortly after die exit. Blends containing 10 g/100 g RPC achieved the highest initial sectional growth followed by substantial short-term shrinkage, but the diameter increased over the next 24 h. The high initial expansion was associated with the potential solubilization of inert rapeseed fibers, conferring much greater water binding and holding capacity and thus promoting swelling at the die exit. The large shrinkage rate in blends with 10 g/100 g RPC was linked to the greater elasticity observed in post-extrusion rheological analysis, initiating elastic recoil after die exit. Blends containing 40 g/100 g RPC exhibited no initial growth, but increased in diameter over a period of 24 h.

Our study revealed that it is important to monitor the initial growth and shrinkage rate of extrudates in addition to the final expansion in order to draw correlations with underlying rheological and/or physicochemical material properties. We also confirmed that that the closed cavity rheometer is a powerful tool that can be used to correlate the rheological properties of biopolymers with the expansion mechanism.

## Figures and Tables

**Table 1:** Chemical composition and particle size of rapeseed press cake (RPC), rapeseed peel (RP), maize starch (MS) and the mixtures of starch with 10 and 40 g/100 g (w.b.) RPC. DM = dry matter.

Raw Material (g/100 g)	DM (g/100 g)	Protein × 6.25 (%DM)	Lipid (%DM)	Raw Fiber (%DM)	Ash (%DM)	Starch (%DM)	Particle Size d_50.3_ (µm)
RPC	95.1 ± 0.03	38.2 ± 0.30	23.4 ± 0.90	4.7 ± 0.03	7.3 ± 0.02	3.00 ± 0.02	261.1 ± 4.5
RP	93.6 ± 0.18	15.7 ± 0.08	25.5 ± 1.12	29.4 ± 0.31	4.1 ± 0.03	4.39 ± 0.03	418.9 ± 15.9
MS	92.3 ± 0.23	n.a.	n.a.	n.a.	n.a.	99.58 ± 0.01	
Calculated Chemical Composition (g/100 g)							
50MS/40RPC	90.86 ± 0.12	16.0 *	15.6 *	3.4 *	n.a.	49.98 ± 0.00	n.a.
70MS/10RPC	91.23 ± 0.11	5.4 *	14.9 *	3.4 *	n.a.	69.25 ± 0.01	n.a.

* Calculated based on the analyzed chemical composition of raw materials. n.a. = not analyzed.

**Table 2:** Extruder response parameters (pressure at the die, product temperature, torque and specific mechanical energy input (SME)) as a function of barrel temperature (T_Barrel_ = 100, 120, 140 °C) of maize starch (MS) blended with 10 or 40 g/100 g (w.b.) rapeseed press cake (RPC) extruded at a moisture content of 24 g/100 g (d.m.).

Sample	Barrel Temperature T_Barrel_ (°C)	Pressure at the Die (bar)	Product Temperature (°C)	Torque (%)	SME (Wh/kg)
MS	100	5.88 ± 0.62 ^a^	91.87 ± 0.83 ^a^	29.77 ± 0.48 ^a^	12.16 ± 0.2 ^a^
MS/RPC10	100	15.07 ± 2.50 ^b^	111.34 ± 1.56 ^b^	17.93 ± 1.63 ^b^	6.94 ± 0.63 ^b^
MS/RPC40	100	8.47 ± 0.79 ^c^	105.98 ± 0.45 ^c^	11.84 ± 0.71 ^c^	4.66 ± 0.23 ^c^
MS	120	5.66 ± 0.07 ^a^	119.00 ± 0.50 ^a^	30.73 ± 0.41 ^a^	12.75 ± 0.17 ^a^
MS/RPC10	120	13.12 ± 3.73 ^b^	121.63 ± 1.63 ^a^	13.47 ± 1.40 ^b^	5.22 ± 0.45 ^b^
MS/RPC40	120	6.99 ± 0.11 ^c^	114.07 ± 0.13 ^b^	11.12 ± 0.16 ^b^	4.38 ± 0.06 ^b^
MS	140	9.42 ± 0.12 ^a^	135.47 ± 2.47 ^a^	27.43 ± 0.58 ^a^	11.67 ± 3.7 ^a^
MS/RPC10	140	11.09 ± 3.92 ^b^	131.38 ± 1.32 ^a^	12.44 ± 1.68 ^b^	4.81 ± 0.65 ^b^
MS/RPC40	140	4.36 ± 0.67 ^c^	124.14 ± 0.29 ^b^	9.44 ± 0.28 ^c^	3.71 ± 0.11 ^b^

Mean values with different superscript letters within one column and barrel temperature indicate significant differences (*p* < 0.05) based on a one-way analysis of variance (ANOVA). When appropriate, means are compared using Tukey’s honest significance test.

**Table 3:** Effect of barrel temperature (T_Barrel_ = 100, 120, 140 °C) and the addition of rapeseed press cake (RPC) (0, 10, 40 g/100 g (w.b.)) to maize starch (MS) extruded at 24 or 29 g/100 g (d.m.) moisture content on dry matter content (g/100 g) after drying extrudates for 24 h at 40 °C.

Blend	Dry Matter after Drying (g/100 g)	Dry Matter after Drying (g/100 g)
	M_melt_ = 24 g/100 g	M_melt_ = 29 g/100 g
T_Barrel_ = 100 °C		
MS	95.45 ± 0.89 ^a^	90.00 ± 1.10 ^a^
MS/RC10	97.18 ± 1.29 ^b^	92.17 ± 0.94 ^b^
MS/RC40	95.94 ± 1.14 ^a^	92.45 ± 1.64 ^b^
T_Barrel_ = 120 °C		
MS	93.22 ± 1.76 ^a^	90.40 ± 1.22 ^a^
MS/RC10	92.56 ± 0.98 ^a^	92.49 ± 1.51 ^b^
MS/RC40	93.97 ± 0.87 ^a^	91.47 ± 2.11 ^b^
T_Barrel_ = 140 °C		
MS	91.34 ± 2.22 ^a^	89.90 ± 0.08 ^a^
MS/RC10	96.36 ± 2.15 ^b^	92.39 ± 1.75 ^b^
MS/RC40	93.69 ± 0.99 ^c^	90.97 ± 1.35 ^a^

Mean values with different superscript letters within one column and barrel temperature indicate significant differences (*p* < 0.05) based on a one-way analysis of variance (ANOVA). When appropriate, means were compared using Tukey’s honest significance test.

## Abbreviations

d.m.	Dry matter (g/100g)
LEI	Longitudinal expansion index
n.a.	Not analysed
M_melt_	Moisture content of melt (g/100 g)
M_dry_	Mass flow rate of dry feed (kg/h)
MS	Maize starch
RO	Rapeseed oil
RP	Rapeseed peel
RPC	Rapeseed press cake
SEI	Sectional expansion index
SME	Specific mechanical energy (Wh/kg)
T	Temperature (°C)
T_Barrel_	Barrel temperature (°C)
T_M_	Measurement temperature (°C)
T_Pre_	Pre-treatment temperature (°C)
VEI	Volumetric expansion index
WAI	Water absorption index
w.b.	Wet basis (g/100 g)
WSI	Water solubility index
$\dot{\gamma }$_Pre_	Pre-treatment shear stress (s^−1^)
$\dot{\gamma }$_M_	Measurement shear stress (s^−1^)
